# Herbal Products: Benefits, Limits, and Applications in Chronic Liver Disease

**DOI:** 10.1155/2012/837939

**Published:** 2012-09-06

**Authors:** Anna Del Prete, Antonella Scalera, Maddalena Diana Iadevaia, Agnese Miranda, Claudio Zulli, Laura Gaeta, Concetta Tuccillo, Alessandro Federico, Carmelina Loguercio

**Affiliations:** Department of Clinical and Experimental Medicine, Interuniversity Research Centre on Food, Nutrition and the Gastrointestinal Tract (CIRANAD), 2nd University of Naples, 80131 Naples, Italy

## Abstract

Complementary and alternative medicine soughts and encompasses a wide range of approaches; its use begun in ancient China at the time of Xia dynasty and in India during the Vedic period, but thanks to its long-lasting curative effect, easy availability, natural way of healing, and poor side-effects it is gaining importance throughout the world in clinical practice. We conducted a review describing the effects and the limits of using herbal products in chronic liver disease, focusing our attention on those most known, such as quercetin or curcumin. We tried to describe their pharmacokinetics, biological properties, and their beneficial effects (as antioxidant role) in metabolic, alcoholic, and viral hepatitis (considering that oxidative stress is the common pathway of chronic liver diseases of different etiology). The main limit of applicability of CAM comes from the lacking of randomized, placebo-controlled clinical trials giving a real proof of efficacy of those products, so that anecdotal success and personal experience are frequently the driving force for acceptance of CAM in the population.

## 1. Introduction

Complementary and alternative medicine (CAM) therapies sought and encompass a wide range of approaches, including two broad categories: exogenous chemicals such as herbal supplements, vitamins, or plant extract, and natural or self-therapies (NST) techniques including relaxation, meditation, prayer, hypnosis, biofeedback, or physical strengthening [1].

The use of herbal medicine began in ancient China at the time of Xia dynasty and in India during the Vedic period [2]. With the revolution of the natural sciences and evidence-based medicine, the divide between Western and Eastern medicines appeared to widen, with CAM reaching an increasing popularity in western countries through years (from 34% of the population in 1990 to 48% in 2004) [3]. 

The age-old system of herbal medicine is being revived by day-to-day practice for its long-lasting curative effect, easy availability, natural way of healing, and less side-effects, so that today herbal medicines are gaining importance and expanding throughout the world [4]. 

The widespread use of CAM is emphasized among people with chronic disease, since it promotes greater personal control over health decision, empowers people to manage their chronic condition, and helps to avoid dissatisfaction often associated with conventional health care [5]. 

CAM is believed to be safer and better than standard medical practice because they are “natural” or are based on a religious, philosophical or a strongly felt concept of “wellness” and health. Treatments with herbal medicine concentrate on reestablishing or reinforcing natural healing processes and wellness [6]. 

Despite increasing popularity, communication about the use of CAM between physicians and patients is limited: most physicians know little about CAM and patients avoid discussing CAM because they fear being received with indifference [7]. 

Moreover, physicians used to focus attention on potential toxicities, even though identification of toxicity from herbal preparations is often difficult, because patients generally self-medicate with these and may withhold this information. Toxic hepatitis is the most common adverse reaction resulting from the use of CAM [8], often associated with the concomitant consumption of hepatotoxic ingredients such as acetaminophen and nonsteroidal anti-inflammatory agents or with hepatotoxicity of herbal ingredients themselves [9]. 

Physicians and health care providers need to become familiar with these products and to recognize potential interaction between conventional drugs and herbals, considering their actual diffusion [10]. 

Botanical medicines have been used traditionally by herbalists and indigenous healers worldwide for the prevention and treatment of liver disease. Clinical research in this century has confirmed the efficacy of several plants in the treatment of liver disease, so the fact that the patients with chronic liver disease seek primary or adjunctive herbal treatment is not surprising.

Particularly, silymarin (an extract of milk thistle) is the most popular product taken by subjects with liver disease and especially by those with hepatitis C virus infection [11]. Seeff et al. [12] found that 41% of outpatients with diagnosis of liver disease had used some form of CAM. Herbal products are often used to improve well-being and quality of life [13] and to ameliorate side effects in patients on antiviral treatment, as fatigue, irritability, and depression: lessening of these symptoms might permit a higher compliance and avoid the need to limit the dose and finally withdraw interferon. 

A systematic review about the use of CAM in chronic hepatitis C has been conducted by Coon and Ernst [14], The authors cited fourteen randomized clinical trials considering the combined use of herbal products and interferon-alfa during antiviral treatment. Although difficulty in extrapolation and interpretation of results because of different methodological limits of the considered studies, the authors found that several herbal products and supplements (vitamin E, thymic extract, zinc, traditional Chinese medicine, Glycyrrhiza glabra, and oxymatrine) could exert potential virological and biochemical effects in the treatment of chronic hepatitis C infection, as a greater clearance of HCV-RNA and normalization of liver enzymes. 

As shown in various studies [15, 16], the use of CAM could be predicted by social, cultural, and geographic factors: sex, age, higher education level, or marriage status of patients are associated with a different use of herbal products. 

The aim of this study is to describe the potential role, benefits, and limits of some of known widespread herbal products in chronic liver disease. We conducted an updated research on Pubmed and Medline in order to refer to more recent articles about this issue. 

## 2. Quercetin 

### 2.1. Definition, Pharmacokinetics, and Biological Aspects

Quercetin is one of the major flavonoids, which represent a class of naturally occurring polyphenolic compounds, ubiquitously present in photosynthesising cells. The intake of flavones and flavonols is determined as 23-24 mg/day and quercetin, the main flavonol present in our diet, represents 70% of this intake. Quercetin is found in fruits (apple) and vegetables, especially onions [17].

Various ways of supplementing quercetin are possible, including a pure supplement or a diet intervention using a food component with a high quercetin content. Supplement usually contains only the aglycon form of quercetin, whereas a food component normally comprises high amounts of various quercetin derivatives that might have a better biological availability than the aglycon itself. Another advantage of a dietary supplementation versus a “conventional” supplement might be a better compliance, especially in long-term use [18]. 

The absorption of quercetin is considerably enhanced by its conjugation with a sugar group. After their facilitated uptake by means of carrier-mediated transport, quercetin glycosides often become hydrolysed by intracellular *β*-glucosidases. After absorption, quercetin becomes metabolized in various organs including the small intestine, colon, liver, and kidney. Metabolites formed in the small intestine and liver are mainly the result of phase II metabolism by biotransformation enzymes and, therefore, include the methylated, sulphated, and glucuronidated forms. Moreover, bacterial ring fission of the aglycon occurs in both small intestine and colon, resulting in the breakdown of the backbone structure of quercetin and the subsequent formation of smaller phenolics [19]. 

Quercetin appears to have many beneficial effects on human health, including cardiovascular protection, anticancer activity, antiulcer and antiallergy activity, cataract prevention, and anti-inflammatory effects. Quercetin has been shown to be an excellent *in vitro* antioxidant. Within the flavonoid family, it is the most potent scavenger of ROS (reactive oxygen species), including O_2_ [20]. 

### 2.2. Quercetin and Alcoholic Liver Disease (ALD)

The pathogenesis and progression of ALD are associated with free radical injury and oxidative stress, which could be partially attenuated by antioxidants and free radical scavengers. Lipid metabolism disorder and oxidative stress play an important role on the development and progression of ALD, and mitochondria compartment is presumed to be the main source and susceptible target of intracellular ROS. The hypothesis that quercetin could prevent ethanol-induced oxidative damage in hepatocytes has been investigated. 

In animal studies, prophylaxis with quercetin-ameliorated ethanol-stimulated mitochondrial dysfunction manifested by decreased membrane potential and by induced permeability transition though suppressing glutathione depletion, enzymatic inactivation of manganese superoxide dismutase, and glutathione peroxidase, ROS overgeneration, and lipid peroxidation in mitochondria. Quercetin, thus, may protect rat, especially hepatic mitochondria, from chronic ethanol toxicity through its hypolipidemic effect and antioxidative role, highlighting a promising preventive strategy for ALD by naturally occurring phytochemicals [21, 22]. 

Quercetin tends to downregulate the ethanol-induced expression of glutathionine peroxidase 4 (GPX4). Furthermore, it tends to reduce the expression of SOD2 induced by ethanol, to downregulate the expression of Gadd45b at the presence of ethanol, which could permit to explain DNA demethylation associated with the upregulation of gene expression in experimental ALD [23]. 

Another study evaluated the effect of quercetin on the parameters classically associated with alcohol liver injury, as lactate dehydrogenase (LDH), aspartate transaminase (AST), malondialdehyde (MDA), glutathione (GSH), superoxide dismutase (SOD), and catalase (CAT) in order to address the alterations of cell damage and antioxidant state after quercetin intervention; the ethanol-intoxicated (100 mM for 8 h) rat primary hepatocytes were simultaneously treated, pretreated (2 h) and posttreated (2 h) with quercetin. The toxic insult of ethanol on hepatocytes was challenged by quercetin and biochemical parameters almost returned to the level of control group when hepatocytes were treated with quercetin at the dose of 50muM for 2–4 h before ethanol exposure [24]. 

A recent study elucidates also a neuroprotective effect of quercetin in alcohol-induced neuropathy through modulation of membrane-bound inorganic phosphate enzyme and inhibition of release of oxidoinflammatory mediators, such as malondialdehyde (MDA), myeloperoxidase (MPO) MPO, and nitric oxide (NO) [25]. 

In conclusion, pretreatment with quercetin provided protection against ethanol-induced oxidative stress in hepatocytes and may be used as a new natural drug for the prevention and/or treatment of ALD. Antioxidants significantly reduced the oxidative stress induced by ethanol intoxication, increased membrane integrity, and also increased organ regeneration [26]. 

### 2.3. Quercetin and Nonalcoholic Fatty Liver Disease (Nafld)

Nonalcoholic fatty liver disease (NAFLD) is the most common cause of chronic liver disease in the United States. It represents the hepatic manifestation of metabolic derangements, known as metabolic syndrome, with insulin resistance playing the major role. Nonalcoholic fatty liver disease includes a variety of histological conditions (ranging from liver steatosis and steatohepatitis, to fibrosis and hepatocarcinoma), all characterized by an increased accumulation/deposition of fat within the liver and associated with alterations in hepatic and systemic inflammatory state. Hepatocytes of a primary cell culture that are exposed to high glucose, insulin, and linoleic acid concentration respond with lipid accumulation, oxidative stress up to cell death. 

Regarding the role of quercetin in NAFLD, it has been show that in mice fed with a Western diet chronic dietary intake of quercetin reduces liver fat accumulation and improves systemic parameters related to metabolic syndrome, probably mainly through decreasing oxidative stress and reducing expression of genes related to steatosis (as PPAR*α*) [27]. 

Another study was aimed to examine the hypoglycemic and insulin-sensitizing capacity of onion peel extract (OPE, containing a high content of quercetin) in high fat diet/streptozotocin-induced diabetic rats and to elucidate the mechanism of its insulin-sensitizing effect. OPE might improve glucose response and insulin resistance associated with type-2 diabetes by alleviating metabolic dysregulation of free fatty acids, by suppressing oxidative stress, upregulating glucose uptake at peripheral tissues, and/or downregulating inflammatory gene expression in liver. Moreover, in most cases, OPE showed greater potency than pure quercetin equivalent. These findings provide a basis for the use of onion peel to improve insulin insensitivity in type-2 diabetes [28]. 

In hepatocytes from normal rats, the decrease in de novo fatty acid and TAG synthesis induced by quercetin represent a potential mechanism contributing to the reported hypotriacylglycerolemic effect of this agent [29]. 

The hepatic response to chronic noxious stimuli may lead to liver fibrosis and to preneoplastic cirrhotic liver. Fibrogenic cells activate in response to a variety of cytokines, growth factors, and inflammatory mediators. The involvement of members of the epidermal growth factor family in this process has been suggested. Amphiregulin is an epidermal growth factor receptor (EGFR) ligand, specifically induced upon liver injury. Recent study investigated the effects of quercetin on the amphiregulin/EGFR signal and on the activation of downstream pathways leading to cell growth: quercetin-ameliorated activation of survival pathways and downregulated the expression of genes related to inflammation and precancerous conditions. Suppression of amphiregulin/EGFR signals may contribute to this effect [30]. 

### 2.4. Quercetin and Hepatitis C Virus (HCV)

Phytochemicals exert antiviral activity and may play a potential therapeutic role in hepatitis C virus (HCV) infection: these aspects were investigated by several studies. 

Park et al. [31] investigated about the antiviral activity in HCV-infected patients of derivates of 7-*O*-arylmethylquercetins. Only five quercetin derivatives showed selective antiviral activity in HCV replicon cell-based assay. 

Recent study studied the quercetin as a potential nontoxic anti-HCV agent in reducing viral production by inhibiting both NS3 and heat shock proteins (that are essential for HCV replication). It was found that quercetin inhibit NS3 activity in a specific dose-dependent manner *in vitro *catalysis assay. Moreover, as analyzed in the subgenomic HCV RNA replicon system, quercetin seemed to exert adjunctive effect: to inhibit HCV RNA replication and production in the HCV infectious cell culture system (HCVcc), as analyzed by the focus-forming unit reduction assay and HCV RNA real-time PCR. The inhibitory effect of quercetin was also obtained when using a model system in which NS3-engineered substrates were introduced in NS3-expressing cells, providing evidence that inhibition *in vivo *could be directed to NS3 and does not involve other HCV proteins. This work demonstrates that quercetin has a direct inhibitory effect on the HCV NS3 protease [32]. 

### 2.5. Quercetin and Hepatitis B Virus (HBV)

Few recent paper discuss about the effects of quercetin on HBV replication and the importance of a glucoside in the structure of quercetin derivatives to exercise a biological action against HBV replication. 

Thirteen flavone glucosides from the herb of Euphorbia humifusa were isolated. Among them, five compounds including apigenin-7-O-*β*-D-glucopyranoside (2), apigenin-7-O-(6′′-O-galloyl)-*β*-D-glucopyranoside (3), luteolin-7-O-*β*-D-glucopyranoside (7), luteolin-7-O-(6′′-O-trans-feruloyl)-*β*-D-glucopyranoside (8), and luteolin-7-O-(6′′-O-coumaroyl)-*β*-D-glucopyranoside (9) showed anti-HBV activity *in vitro*. Anti-HBV activity was closely related to the parent structure of these compounds (agigenin > luteolin > quercetin) as well as to the number of glucoside (flavone monoglucoside > flavone diglucoside). The structure of these agent also influences their cytotoxicity (flavone > flavone monoglucoside > flavone diglucoside). In addition, the substitution of acyl group on glucoside may be important to keep their anti-HBV activities (galloyl > feruloyl > coumaroyl) [33]. Quercetin did not show activities against HBeAg secretion: this limit might be due to the absence of the saccharide group in their structures [34]. 

## 3. Curcumin 

### 3.1. Definition, Pharmacokinetics, and Biological Aspects

Curcumin is a low-molecular-weight polyphenol derived from the rhizomes of turmeric (curcuma longa). It represents a yellow pigment widely used as a coloring agent and spice in many foods. It has various beneficial pharmacological effects including antioxidant, anti-inflammatory, anticarcinogenic, hypocholesterolemic, antibacterial, antispasmodic, anticoagulant, and hepatoprotective activities [35].

Phase I clinical trials have shown that curcumin is safe even at high doses (12 g/day) in humans but exhibit poor bioavailability. Despite the promising biological effects of curcumin, low plasma and tissue levels of curcumin due to its poor oral bioavailability and absorption, rapid metabolism and systemic elimination in both rodents and humans [36] may be responsible for the unfavorable pharmacokinetic of this molecule. To improve the bioavailability of curcumin, numerous approaches have been undertaken. These approaches involve, first, the use of adjuvant like piperine that interferes with glucuronidation; second, the use of liposomal curcumin; third, curcumin nanoparticles; fourth, the use of curcumin phospholipid complex; fifth, the use of structural analogues of curcumin (e.g., EF-24) [35, 36].

### 3.2. Curcumin and Alcohol Liver Disease (ALD)

Liver fibrosis can be explained with an increased deposition of extracellular matrix (ECM). Chronic alcohol abuse is one of the main causes of liver fibrosis. Ingestion of polyunsaturated fatty acids (PUFAs) together with alcohol can aggravate the toxicity of alcohol. The degree of abnormal ECM degradation depends on the ratio of active matrix metalloproteinases (MMPs) and tissue inhibitors of metalloproteinases (TIMPs). A recent work studied the influence of bis-desmethoxy curcumin analog (BDMC-A) on the expression of MMPs and TIMPs during alcohol and DeltaPUFA-induced liver toxicity. Administration of BDMC-A significantly decreased the levels of collagen and TIMPs and positively modulated the expression of MMPs. From this study, we can conclude that BDMC-A influences MMPs, TIMPs expression and that it acts as an efficient anti-fibrotic agent [37]. 

It has been demonstrated the potential protective effect of curcumin pretreatment against ethanol-induced hepatocytes oxidative damage, with emphasis on heme oxygenase-1 (HO-1) induction. Ethanol exposure resulted in a sustained malondialdehyde (MDA) elevation, glutathione (GSH) depletion, and evident release of cellular lactate dehydrogenase (LDH) and aspartate aminotransferase (AST), which was significantly ameliorated by curcumin pretreatment. In addition, dose- and time-dependent induction of HO-1 was involved in such hepatoprotective effects by curcumin. Curcumin exerts hepatoprotective properties against ethanol involving HO-1 induction, which provide new insights into the pharmacological targets of curcumin in the prevention of alcoholic liver disease [38]. 

To study the mechanism of curcumin-attenuated inflammation and liver pathology in early stage of alcoholic liver disease, female Sprague-Dawley rats were divided into four groups and treated with ethanol or curcumin via an intragastric tube for 4 weeks. A control group was treated with distilled water and an ethanol group was treated with ethanol (7.5 g/kg bw). Treatment groups were fed with ethanol supplemented with curcumin (400 or 1 200 mg/kg bw). The liver histopathology in ethanol group revealed mild-to-moderate steatosis and mild necroinflammation. Hepatic MDA, hepatocyte apoptosis, and NF-kappaB activation increased significantly in ethanol-treated group when compared with control. Curcumin treatments resulted in improving of liver pathology, decreasing the elevation of hepatic MDA, and inhibition of NF-kappaB activation. The 400 mg/kg bw of curcumin treatment also revealed a trend of decreased hepatocyte apoptosis. However, the results of SOD activity, PPARgamma protein expression showed no difference among the groups. In conclusion, curcumin improved liver histopathology in early stage of ethanol-induced liver injury by reduction of oxidative stress and inhibition of NF-kappaB activation [39].

### 3.3. Curcumin and Nafld

In animal study, it has been demonstrated the capacity of curcumin to improve liver histology of NAFLD. The NASH model was induced by high-fat diet combined with carbon tetrachloride. These rats were successively treated with curcumin and curcumin derivative. The results showed a remarkable reduction in serum ALT (U/L), AST (U/L) in rats treated with curcumin derivatives. The degrees of fibrosis were also significantly alleviated. The reduction of the gene transcriptions of TNF-alpha, NF-kappaB, and HMG-CoA was the mechanism proposed by wich curcumin exercise its beneficial effects in NASH. The results of this study indicate, moreover, that the water-soluble curcumin derivative displays superior bioavailability to the parent curcumin, which can effectively improve the lipid metabolism and delay the progression of hepatic fibrosis in rats with steatohepatitis [40]. 

Interestingly, it also demonstrates that the activating effect of LDL can be reversed by curcumin. The hypocholesterolemic action of curcumin was reported by numerous studies: curcumin seems to reduce serum cholesterol concentrations by increasing hepatic expression of LDL receptors, by blocking LDL oxidation, increasing bile acid secretion, and faecal excretion of cholesterol, repressing the expression of genes involved in cholesterol biosynthesis and protecting against liver injury and fibrogenesis [41, 42]. Curcumin induces apoptosis and blocks proliferation of hepatic stellate cells (HSCs), and, via activation of PPAR*γ*, inhibits extracellular matrix formation, downregulates the expression of LDL receptors, induces *SREBP-1c*,and increases the fat-storing capacity of HSC, and it may thereby restore this “protective” functionality, proving of therapeutic usage in preventing liver steatosis and fibrosis [43]. 

An Italian study tested whether the administration of curcumin limits fibrogenic evolution in a murine model of nonalcoholic steatohepatitis. They demonstrated that curcumin decreased the intrahepatic gene expression of monocyte chemoattractant protein-1, CD11b, procollagen type I, and tissue inhibitor of metalloprotease (TIMP)-1, together with protein levels of alpha-smooth muscle actin, a marker of fibrogenic cells. In addition, curcumin reduced the generation of reactive oxygen species in cultured HSCs and inhibited the secretion of TIMP-1 both in basal conditions and after the induction of oxidative stress. This study so proposed that curcumin administration effectively limits the development and progression of fibrosis in mice with experimental steatohepatitis and reduces TIMP-1 secretion and oxidative stress in cultured stellate cells [44]. 

High consumption of dietary fructose is an important contributory factor in the development of hepatic steatosis in insulin or leptin resistance. The effects of curcumin on fructose-induced hypertriglyceridemia and liver steatosis and its preventive mechanisms in rats have been investigated. Curcumin reduced serum insulin and leptin levels in fructose-fed rats and it protects against fructose-induced hypertriglyceridemia and hepatic steatosis by inhibition of PTP1B (hepatic protein tyrosine phosphatase 1B) and subsequent improvement of insulin and leptin sensitivity in the liver of rats. This PTP1B inhibitory property may represent a promising role for curcumin to treat fructose-induced hepatic steatosis induced by hepatic insulin and leptin resistance [45, 46].

### 3.4. Curcumin and HCV

Curcumin is known to exert anti-viral activity against influenza virus, adenovirus, coxsackievirus, and the human immunodeficiency virus [47, 48]. However, it remains to be determined whether curcumin can inhibit the replication of hepatitis C virus (HCV). A study showed that curcumin decreases HCV gene expression via suppression of the Akt-SREBP-1 activation, not by NF-kappaB pathway. The combination of curcumin and IFN-alpha exerted profound inhibitory effects on HCV replication. These results indicate that curcumin can suppress HCV replication *in vitro* and may be potentially useful as novel anti-HCV reagents [49].

### 3.5. Curcumin and HBV

Hepatitis B virus (HBV) infects the liver and uses its cell host for gene expression and propagation. Therefore, since targeting host factors is essential for HBV gene expression, it could represent a potential anti-viral strategy. 

Curcumin treatment could complement the antiviral activity of the nucleotide/nucleoside analogues, which are considered as the gold standards for anti-HBV therapy. The combination of Lamivudine and curcumin treatments resulted in an enhanced suppression of HBV expression by up to 75%, as compared to nontreated cells. These results suggest that curcumin may work synergistically with the current anti-HBV nucleotide/nucleoside analogous, and that this combination may result in a better suppression of HBV. Moreover, curcumin inhibits HBV gene expression and replication, by downregulating PGC-1alpha, a starvation-induced protein that initiates the gluconeogenesis cascade and that has been shown to robustly coactivate HBV transcription [50, 51]. 

## 4. Silymarin and Its Derivates 

### 4.1. Definition, Pharmacokinetics, and Biological Aspects

Silybum marianum, also known as milk thistle, is a member of Asteraceae family and is well recognized as a hepatoprotective herbal medicine. Silymarin is a lipophilic extract of the milk thistle seeds. It is composed of three isomers of flavonolignans (silybin, silydianin, and silychristin), and two flavonoids (taxifolin and quercetin). 

Silymarin has revealed poor absorption, rapid metabolism, and ultimately poor oral bioavailability. For optimum silymarin bioavailability, issues of solubility, permeability, metabolism, and excretion must be addressed. An array of methods have been described in recent years that can improve its bioavailability, including complexation with *β*-cyclodextrins, solid dispersion method, formation of microparticles and nanoparticles, self-microemulsifying drug delivery systems, micelles, liposomes, and phytosomes [52]. 

Silymarin possesses various pharmacological activities, including hepatoprotective, antioxidant, anti-inflammatory, anticancer, and cardioprotective effects. *Silybum marianum *is the most well-researched plant in the treatment of liver diseases. Silymarin has been shown to have a variety of anti-inflammatory effects on liver, including mast cell stabilization, inhibition of neutrophil migration, and Kupffer cell inhibition [53]. Silymarin is commonly prescribed in cases of cirrhosis or viral hepatitis. 

### 4.2. Sylimarin and Its Derivates in ALD

Hepatocyte models were proposed as a platform for screening of herbal component against ethanol hepatotoxicity. Nanosilibinin, for the first time, found to perform significant protection against ethanol-induced hepatotoxicity while silibinin in normal particles could not inhibit such toxicity. This protection of nanosilibinin might be related to its high bioavailability compared to normal insoluble silibinin and could act as an antioxidative and antisteatosis agent against ethanol-induced hepatotoxicity [54]. 

The affect of silymarin on the levels of serum ALT and GGT in ethanol-induced hepatotoxicity in albino rats has also been tested. Eighteen male albino rats aged 6–8 weeks, weighing 150–200 gm, were divided into 3 groups of 6 rats each: group A as control, group B as rats taking ethanol at a dose of 0.6 mL (0.5 gm)/100 gm/day, and group C taking ethanol and silymarin at a dose of 0.5 gm/100 gm/day and 20 mg/100 gm/day, respectively, for 8 weeks. Silymarin tends to normalize liver function test in alcoholic liver disease [55]. 

Acute ethanol administration caused prominent hepatic microvesicular steatosis with mild necrosis and an elevation of serum ALT activity, induced a significant decrease in hepatic glutathione in conjunction with enhanced lipid peroxidation (oxidative stress) and increased hepatic TNF (necrosis factor-alpha) production. Supplementation with a standardized silymarin with its both antioxidant and anti-inflammation properties decreases TNF production and attenuated these adverse changes induced by acute ethanol administration. In view of its nontoxic nature, it may be developed as an effective therapeutic agent for alcohol-induced liver disease by its antioxidative stress and anti-inflammatory features [56]. 

### 4.3. Slymarin and Its Derivate in Nafld

Silymarin showed a significant hypocholesterolemic effect compared to the diet model with high fat-diet (HFD). Moreover, silymarin significantly reduces TG levels compared to HFD group. 

The elevation of transaminases usually reflects necrosis of hepatocytes: with silymarin, ALT levels (a specific index of hepatic necrosis) were particularly reduced [57]. 

Assuming that oxidative stress leads to chronic liver damage, Loguercio et al. conducted a study about the antioxidant activity of silybin conjugated with vitamin E and phospholipids. Eighty-five patients were divided into 2 groups: those affected by nonalcoholic fatty liver disease (group A) and those with HCV-related chronic hepatitis associated with nonalcoholic fatty liver disease (group B), nonresponders to antiviral treatment. The treatment consisted of silybin/vitamin E/phospholipids. After treatment, group A showed a significant reduction in ultrasonographic scores for liver steatosis. Liver enzyme levels, hyperinsulinemia, and indexes of liver fibrosis showed an improvement in treated individuals. A significant correlation among indexes of fibrosis, body mass index, insulinemia, plasma levels of transforming growth factor-beta, tumor necrosis factor-alpha, degree of steatosis, and gamma-glutamyl transpeptidase was observed. Our data suggest that silybin conjugated with vitamin E and phospholipids could be used as a complementary approach in the treatment of patients with chronic liver damage [58]. 

### 4.4. Sylimarin and Its Derivates in HCV Infection

Silymarin and its purified flavonolignans have been recently demonstrated to inhibit hepatitis C virus (HCV) infection, both *in vitro* and *in vivo*. 

Silymarin showed antiviral effects against hepatitis C virus cell culture (HCVcc) infection that included inhibition of virus entry, RNA and protein expression, and infectious virus production. Silymarin did not block HCVcc binding to cells but inhibited the entry of several viral pseudoparticles (pp), and fusion of HCVpp with liposomes. Silymarin also blocked cell-to-cell spread of virus [59]. 

Pegylated interferon (PEG-IFN) plus Ribavirin therapy is the current treatment for the patient with chronic hepatitis C. The main goal of therapy is to achieve a sustained virological response (SVR is defined as undetectable HCV-RNA in peripheral blood determined with the most sensitive polymerase chain reaction technique 24 weeks after the end of treatment). This goal is practically equivalent with eradication of HCV infection and cure of the underlying HCV-induced liver disease. This therapy is effective only in half of patients, because of important side-effects, resistance, and high cost related to therapy. Silymarin inhibits both HCV RNA (in a dose-dependent manner) and HCV core expression thanks to its direct effect against HCV 3a core or activation of JAK/STAT pathways, resulting in inhibition of HCV core gene, by phosphorylation of Stat1 on tyrosine and serine [60]. 

Silymarin but not silibinin inhibited genotype 2a NS5B RNA-dependent RNA polymerase (RdRp) activity at concentrations 5 to 10 times higher than required for anti-HCVcc effects. Furthermore, silymarin had inefficient activity on the genotype 1b. Although inhibition of *in vitro* NS5B polymerase activity is demonstrable, the mechanisms of silymarin's antiviral action appear to include blocking of virus entry and transmission by targeting the host cell [60, 61]. 

In another study, patients with chronic hepatitis C performing silymarin (650 mg/day) for 6 months improved serum HCV-RNA titer, serum aminotransferases (ALT, AST), hepatic fibrosis, and patient's quality of life. After the treatment, nine patients were found with negative HCV-RNA and statistically significant improvement in results of liver fibrosis markers was found only in fibrosis group. 

So, for its antioxidant and anti-inflammatory actions, sylimarin could result useful in reducing hepatic inflammation in chronic liver disease, including HCV-related damage. It has been hypothesized that decreased hepatic inflammation-due to both direct and indirect effects of silymarin in decreasing viral replication has the potential to induce long-term benefit to the infected liver [62]. 

Since oxidative stress may play a pathogenetic role in chronic hepatitis C, and sustained virological response to antiviral therapy is limited in HCV1 genotype infection, a double-blind study was performed in HCV1 patients treated with pegylated interferon + ribavirin in order to assess the efficacy of supplementation with the antioxidant flavonoid silymarin. In the silymarin group, a more rapid decrease in the malondialdehyde level as well as a marked decrease in superoxide dismutase and an increase in myeloperoxidase activity after twelve months of treatment were found. In particular, alanine aminotransferase normalized in 6/16 (versus control 9/16) cases and sustained virological response occurred in 3/16 (versus 7/16) patients [63].

### 4.5. Silymarin and Its Derivates in HBV Infection

Few recent data discuss about the role of silymarin in the hepatitis B. We only reported silymarin beneficial effects in early stages of liver pathogenesis, in preventing and delaying liver carcinogenesis. 

This drug should be considered as a potential chemopreventive agent for HBV-related hepatocarcinogenesis [64]. 

## 5. Betaine 

### 5.1. Definition, Pharmacokinetics, and Biological Aspects

Betaine is a naturally occurring dietary compound that is also synthesized *in vivo* from choline. *In vivo*, betaine acts as a methyl donor for the conversion of homocysteine to methionine and its also functions as an osmolyte. 

### 5.2. Betaine and ALD

Chronic ethanol exposure has been shown both to decrease hepatic concentrations of *S*-adenosylmethionine (SAM) and plasma concentrations of folate in animal and human studies and to increase plasma concentrations of homocysteine and hepatic levels of *S*-adenosylhomocysteine (SAH) [65]. 

In the liver, betaine can transfer its methyl group to homocysteine in order to form methionine. This can result in decreased concentrations of homocysteine and increased concentrations of methionine in the liver, resulting in decreased hepatic concentrations of SAH, whereas the latter can increase hepatic SAM concentrations, which leads to an increased SAM : SAH. An elevated SAM : SAH can trigger a cascade of events leading to formation of proper VLDL, to export of triacylglycerol and attenuation of fatty liver. Increased hepatic concentrations of SAM can activate cystathionine-synthase and lead to upregulation of the *trans-*sulfuration pathway, to increase synthesis of glutathione and attenuate oxidative stress. Thus, betaine can ameliorate ALD by attenuating fatty liver, inflammation, and fibrosis [66]. 

The role of mitochondrial dysfunction in the pathogenesis of alcoholic liver disease has been long documented by multiple laboratories. The dietary supplementation with betaine protects against ethanol-induced loss in oxidative phosphorylation system proteins. Even if the exact mechanism for this protection at the organelle level is not known, betaine shows to preserve the function of the electron transport chain, to maintain the integrity of the liver, and to protect against the development of alcoholic liver injury by preventing NOS_2_ induction and NO generation [67]. Moreover, specific changes are associated with the normalization of hepatic SAM : SAH ratio and maintenance of methylation potential in response to betaine supplementation during chronic ethanol ingestion. 

Chronic alcohol administration increases gut-derived endotoxin in the portal circulation, activating Kupffer cells to produce several proinflammatory cytokines such as tumor necrosis factor-*α* (TNF-*α*) and interleukin (IL)-1. 

Ethanol administration can also lead to the synthesis of Toll-like receptor 4 (TLR4) protein and its gene expression in Kupffer cells, indicating that TLR4 may play a major role in the development of alcohol-induced liver injury. The intragastric ethanol-fed rat model, which reproduces the pathological features of early alcohol-induced liver injury, was used to observe the changes of TLR4 expression and the effect of betaine in alcohol-induced liver injury animal models. 

It has been suggested that betaine can prevent alcohol-induced liver injury effectively and improve liver function. The hepatoprotective mechanism of betaine is probably related to the inhibition of endotoxin/TLR4 signaling pathways. In rats with alcohol-induced liver injury, betaine feeding can decrease the levels of serum ALT, AST, endotoxin, TNF-*α*, IFN-*γ*, and IL-18, reduce the expressions of TLR4, and improve the degree of hepatic steatosis and inflammation in liver tissues. 

In summary, the results of this study show that the expression of TLR4 increased significantly in ethanol-fed rats. Betaine administration can inhibit TLR4 expression, which may be the mechanism of protection from alcoholic liver injury exerted by betaine [68].

### 5.3. Betaine and NAFLD

The role of betaine in the treatment of NASH has been evaluated in human studies. Oral administration of betaine glucuronate in NASH patients for 8 weeks reduced both hepatic steatosis by 25% and hepatomegaly by 8%, and it significantly attenuated serum concentrations of AST, ALT, and glutamyl transferase. Similarly, a marked improvement in the degree of steatosis, necroinflammatory grade, and stage of fibrosis was obtained after treatment with betaine [69]. 

Nonalcoholic fatty liver disease (NAFLD) is a common liver disease, associated with insulin resistance. Betaine treatment would prevent or treat NAFLD in mice. Betaine reduces fasting glucose, insulin, triglycerides, and hepatic fat in mice submitted to a moderate high-fat diet (MHF). Betaine significantly improved insulin resistance and hepatic steatosis. Betaine treatment reversed the inhibition of hepatic insulin signaling in mHF and in insulin-resistant HepG2 cells, including normalization of insulin receptor substrate 1 (IRS1) phosphorylation and of signaling pathways for gluconeogenesis and glycogen synthesis. We can conclude that betaine treatment prevents and treats fatty liver in a moderate high-dietary-fat model of NAFL D in mice [70]. 

Moreover, betaine supplementation alleviated hepatic pathological changes, which were concomitant with attenuated insulin resistance (as shown by improved homeostasis model assessment of basal insulin resistance values and glucose tolerance test) and corrected abnormal adipokine productions (adiponectin, resistin, and leptin). Specifically, betaine supplementation enhanced insulin sensitivity in adipose tissue as shown by improved extracellular signal-regulated kinases 1/2 and protein kinase B activations. In adipocytes, freshly isolated from mice fed a high-fat diet, pretreatment of betaine enhanced the insulin signaling pathway and improved adipokine productions. Further investigation using whole liver tissues revealed that betaine supplementation alleviated high-fat diet-induced endoplasmic reticulum stress response in adipose tissue as shown by attenuated glucose-regulated protein 78/C/EBP homologous protein (CHOP) protein abundance and c-Jun NH2-terminal kinase activation [71]. 

Song et al. showed that betaine significantly attenuated hepatic steatosis induced by high-sucrose diet (animal model), and this change was associated with increased activation of hepatic AMP-activated protein kinase (AMPK) and attenuated lipogenic capability (enzyme activities and gene expression) in the liver [72]. 

### 5.4. Betaine and HCV

The cause of failure of antiviral treatment with standard therapy, that is, pegylated interferon alpha (pegIFNalpha) combined with ribavirin, in half patients, is unknown, but viral interference with IFNalpha signal transduction through the Jak-STAT pathway could be considered. The expression of HCV proteins leads to an impairment of Jak-STAT signaling because of the inhibition of STAT1 methylation. Unmethylated STAT1 is less active since it can be bound and inactivated by its inhibitor, protein inhibitor of activated STAT1 (PIAS1). The treatment of cells with S-adenosyl-L-methionine (AdoMet) and betaine could restore STAT1 methylation and improve IFN alpha signaling. Furthermore, the antiviral effect of IFNalpha in cell culture could be significantly enhanced by the addition of AdoMet and betaine. S-adenosyl-L-methionine (SAMe) and betaine potentiate IFN*α* signaling in cultured cells expressing hepatitis C virus (HCV) proteins and enhance the inhibitory effect of IFN*α* on HCV replicons. SAMe and betaine were found to be safe when used with pegIFN*α*/ribavirin [73]. 

In conclusion, the addition of these drugs to antiviral standard therapy for patients with chronic hepatitis C could overcome the problem of drug resistance [74]. 

Homocysteine, a sulfuric amino acid involved in methionine metabolism, belongs to the group of intracellular thiols. Hyperhomocysteinemia is frequent in the Caucasian and its role in vascular pathology has been clearly established. In hepatology, experimental data in transgenic mice deficient in homocysteine metabolism enzymes have shown the presence of severe liver steatosis with occasional steatohepatitis. In chronic hepatitis C, preliminary data have shown that hyperhomocysteinemia is an independent risk factor for steatosis or even fibrosis. The physiopathological mechanism has now begun to be better understood. On one hand, there is a strong correlation between homocysteine and insulin resistance whatever its etiology. On the other hand, homocysteine has a direct effect on the liver, resulting in overexpression of SREBP-1 and favoring steatosis. It stimulates proinflammatory cytokine secretion such as NF kappa B increasing the risk of NASH. Finally, homocysteine could increase the risk of fibrosis by stimulating TIMP 1. Moreover, hepatitis C virus induces hypomethylation of STAT 1 and could decrease the antiviral activity of interferon. Results from *in vitro* studies have shown that the normalization of STAT 1 methylation by bringing betaine and S Adenosyl Methionine (which belongs to homocysteine cycle) restores the antiviral activity of interferon. Finally, treatment of hyperhomocysteinemia could have favorable consequences in steatohepatites and HCV infection [75, 76]. 

### 5.5. Betaine and HBV

Only few data exist about the role of betaine in histological or clinical effects of hepatitis-B-virus-infected patients. 

## 6. Glycyrrhiza Glabra 

### 6.1. Definition, Pharmacokinetics, and Biological Aspects

Glycyrrhiza glabra (licorice root), a perennial herb cultivated in temperate and subtropical regions of the world and native to Mediterranean region as well as to central and South-Western Asia, belongs to the Leguminosae family, genus Glycyrrhiza [77]. The aqueous extract of this plant contains Glycyrrizin (GL), a conjugate of two molecules of glucuronic acid and one of 18*β*-glycyrrhetic acid (GA) [78] and other substances as flavonoids, hydroxycoumarins and beta-sitosterols [79]. 

Stronger neominophagen C (SNMC) is a product used in Japan for the treatment of acute and chronic hepatitis. This solution is administered intravenously, 80–200 mg/day, for variable periods of time, and contains 0.2% glycyrrhizin, 0.1% cysteine, and 2% glycine in physiological solution. In the United States, glycyrrhizin is available in a multiplicity of nonstandardized oral formulations found over the country [80, 81]. 

GL injected i.v., is partially metabolized to 3-monoglucuronyl-glycyrrhetinic acid (3MGA) in the liver by lysosomal *β*-D-glucuronidase, and GL and 3MGA could be excreted with bile. The biliary-excreted GL and 3MGA are hydrolyzed by intestinal bacteria into GA, which is reabsorbed into the bloodstream. Orally administered GL is enzymatically hydrolyzed to GA by intestinal bacterial flora before absorption into the bloodstream. The circulated GA is further metabolized to 3MGA in the liver by UDP-glucuronyl transferase and then excreted with bile into the intestine [82]. The pharmacokinetic characteristics of i.v. administration of GL in patients with liver disease was studied in a Japanese and European report: SNMC has linear pharmacokinetics up to 200 mg, and steady state in achieved after two weeks of 200 mg doses administrated six times per week [83]. 

Potential interactions may occur with drugs metabolized by CYP450 3A4, although those have not been reported to date [80, 81]. 

Decreases of potassium, sodium retention, worsening of ascites, and hypertension are possible adverse effects due to the 11-hydroxy-steroid dehydrogenase inhibitory activity of GL and GA [84]. However, published data show no increased rate of these side-effects during treatment although documentation of toxicity is poor in most reports [77]. 

The use of GL in acute and chronic hepatitis is due to its hepatoprotective, immunomodulatory, and anti-inflammatory effect. It reduced ischemia/reperfusion (I/R-) induced liver injury [85] and inhibited hight-mobility group box 1 (HMGB1), an inflammatory cytokine that acted in inflammation and organ damage in hepatic I/R-injury [85, 86]. 

Many studies shown that GL attenuated inflammatory responses due to decreased activation of nuclear factor kB (NF-kB) and mitogen-activated protein kinase (MAPK) pathways [87], moreover, inhibited the production of LPS-induced nitric oxide (NO) and tumor necrosis factor-*α* (TNF-*α*), prostaglandin E2, intracellular reactive oxygen species (ROS), proinflammatory interleukins as IL-4, IL-5, IL-6, IL-18, IL-1*β* [88–92], and increased the production of anti-infiammatory interleukins as IL-12 and IL-10 [92]. 

In animal studies, GL inhibits CD4+ T-cell and tumor necrosis factor- (TNF-) mediated cytotoxicity [93], activated NK cells and extrathymic T lymphocyte differentiation [94, 95], and promoted maturation of dendritic cells [92]. 

GL inhibited serum AST and ALT levels and histologically inhibited the infiltration of inflammatory cells and the spreading of degenerative areas of hepatocytes in an animal model of concanavalin A-induced liver injury [96]. 

### 6.2. Glycyrrizin in HCV and HBV Infection

Many studies showed GL have antiviral activity (reviewed in [97]). A mechanism proposed for explain this propriety is the membrane stabilizing effect, as demonstrated in rat hepatocytes incubated with antibody raised against rat liver cell membranes: rat hepatocytes released AST after incubation with antiliver cell antibody in the presence of complement, and the endogenous phospholipase A2 activity was increased, but glycyrrhizin suppressed phospholipase A2 activity and reduced transaminase level [98]. A more recent study confirms this propriety in HIV and Influenza A virus [99]. 

Despite a precedent review [100] evidenced that SNMC acts as an antiinflammatory or cytoprotective drug but does not have antiviral properties, a recent *in vitro* study found that GL inhibit HCV full-length viral particles and HCV core gene expression or function in a dose-dependent manner and had synergistic effect with interferon [101] and a European randomized trial showed biochemical effects of 26-week treatment with SNMC in patients with chronic hepatitis C [102]. 

Glycyrrhizin-modified glycosylation and blocked sialylation of hepatitis B surface antigen (HBsAg) [103]. An *in vitro* study, measuring the release of surface protein (HBsAg) and HBV-DNA in transfected HepG2 2.2.15 cells, showed that this compound had a moderate ability in reducing viral production [104]. 

Long-term clinical trials in Japan and The Netherlands demonstrate that interferon nonresponder patients with chronic hepatitis C and fibrosis stage 3 or 4 have a reduced incidence rate of HCC after glycyrrhizin therapy normalizes ALT levels [105, 106]. Other well-diagnosed studies are needed to better define the role of GL in HBV and HCV-related liver disease. 

### 6.3. Glychyrrizin and Its Protective Role in Hepatic Fibrosis and Steatosis

18 beta-glycyrrhetinic acid (18*α*-GL) can suppress the activation of hepatic stellate cells (HCSs) and induce their apoptosis by blocking the translocation of NF-kappaB into the nucleus, furthermore, it promoted the proliferation of hepatocytes in rats with CCl4-induced liver fibrosis [107]. GA inhibited type I collagen synthesis and progression of liver fibrosis probably through the suppression of collagen gene (COL1A2) promoter [108]. In transgenic mice expressing the HCV polyprotein fed an excess iron diet, SNMC prevented hepatic steatosis: this product attenuated ultrastructural alterations of mitochondria of the liver, activated mitochondrial *β*-oxidation with increased expression of carnitine palmitoyl transferase I and decreased the production of reactive oxygen species. 

Wu et al. found that 18*α*-GL, the biologically active metabolite of GL, prevented FFA-induced lipid accumulation and cell apoptosis in *in vitro* HepG2 NAFLD models and also prevented high-fat-diet-induced hepatic lipotoxicity and liver injury *in vivo* rat NAFLD models. GA stabilized lysosomal membranes, inhibited cathepsin B expression and enzyme activity, inhibited mitochondrial cytochrome c release, and reduced FFA-induced oxidative stress [109]. 

### 6.4. Glycyrrizin and Anticancer Activity in Liver

A recent review revealed that triterpenoids, which are also found in Glycyzirra glabra extract, had antitumor activities. Triterpenoids could induce apoptosis in various cancer cells by activating various proapoptotic signaling cascades. The molecular mechanisms involved include inhibition of various oncogenic and antiapoptotic signaling pathways and suppression or nuclear translocation of transcription factors including NF-*κ*B [110]. In a human hepatoma cell line, the expression of junB mRNA, a tumor suppressor gene, and JUNG protein is highly increased by GL treatment [111]. Zhao et al. studied the *β*-Cyclodextrin/glycyrrhizic acid functionalized quantum dots (*β*-CD/GA-functionalized QDs [112], and found that this drug has proapoptotic effects in hepatocarcinoma cells. *β*-CD/GA-functionalized QDs triggered G0/G1 phase arrest and induced apoptosis through an reactive oxygen species mediated mitochondrial dysfunction pathway.

## 7. Phyllantus spp. 

### 7.1. Definition, Pharmacokinetics, and Biological Aspects

The genus Phyllantus (Euphorbiaceae) consist of about 6500 species in 300 genera, of which 200 are American, 100 African, 70 from Madagascar, and the remaining are Asian and Australian [113]. Many species are used in traditional medicine mainly in India and China to treat several diseases [114] and a morphological analysis of samples of Phyllanthus used in raw drug trade in southern India shown that 76% of the market samples contained P. amarus as the predominant species (>95%) and other five different species, namely, *P. debilis, P. fraternus, P. urinaria, P. maderaspatensis*, and *P. kozhikodianus*, were found in the remaining 24% of the shops [115]. 

P. amarus has been widely studied because it is the most commonly used in the Indian Ayurvedic medicine in the treatment of gastrointestinal and genitourinary diseases. Its main active components are ligans, as phyllanthin and hypophyllanthin, and flavonoids, alkaloids, hydrolysable tannins, polyphenols, triterperens, sterols, and volatile oil [113]. 

Many animal studies evidenced the hepatoprotective activity of P. amarus. The extract enhanced liver and serum alanine transaminase (ALT), aspartate transaminase (AST), alkaline phosphatase (ALP), acid phosphatase (ACP), glutathione-S transferase (GST). Furthermore lipid peroxidation level was significantly reduced in ethanol and CCl4-induced liver disease animal models [116–119]. The administration of P. amarus extract significantly decreased the levels of collagen and tissue inhibitors of matrix metalloproteinases (TIMPs) and positively modulated the expression of matrix metalloproteinases (MMPs) in rats with alcohol and thermally oxidized polyunsaturated fatty acid- (PUFA-) induced hepatic fibrosis [120]. In mice with aflatoxin B1-induced liver damage, P. amarus extract lowered down the content of thiobarbituric acid reactive substances (TBARSs) and enhanced the reduced glutathione level and the activities of antioxidant enzymes, glutathione peroxidase (GPx), glutathione-S-transferase (GST), superoxide dismutase (SOD), and catalase (CAT) [121]. 

In a recent animal study, it was found a synergistic effect between silymarin and P. amarus, especially with ethanoic extract of P. amarus, due to its higher concentration of phyllanthin in comparison to aqueous extract against CCl-induced nepatotoxicity [122]. 

The hepatoprotective action was studied also in other Phyllanthus species, as P. simplex [123], P. atropurpureus [124], P. acidus [125, 126], P. fraternus [127], P. emblica [128], P. urinaria, and P. maderaspatensis [129, 130], and significant antioxidant and anti-inflammatory activities were found especially in P. simplex extracts because of its high phenolic content [123]. Finally, the antioxidant activity was compared between P. virgatus and P. amarus and was found that the first had higher cytotoxicity, higher free radical scavenging activity, and more inhibition of peroxidation capacity [131]. 

### 7.2. Phyllanthus and Alcohol-Induced Liver Disease

Preclinical studies have shown that Emblica officinalis (P. emblica) protect against ethanol-induced hepatotoxicity (reviewed in [132]). Animal studies showed that the fruit extract improved plasma enzymes level, reduced lipid peroxidation, and restored the enzymatic and nonenzymatic antioxidants level in alcohol-induced liver disease, this action is probably due to tannoid, flavonoid and NO scavenging compounds present in the extract [133–135]. Phyllanthin restored the antioxidant capability of rat hepatocytes including level of total glutathione, and activities of superoxide dismutase (SOD) and glutathione reductase (GR) which were reduced by ethanol [136] and P. amarus-modified alcohol and thermally oxidized PUFA-induced fibrosis in rats because decreased the levels of collagen and TIMPs and positively modulated the expression of MMPs [120]. 

### 7.3. Phyllanthus in HCV and HBV Infection

P. amarus extract was used, in 1988, in a preliminary study involving 37 patients with chronic hepatitis B. 22 of 37 treated patients had cleared the virus 2-3 weeks after the end of the treatment period and only one of 23 placebo treated became HBsAg negative [137]. The mechanism of action appears to be related to the suppressive effect of Phyllanthus extract on HBsAg secretion and HBsAg mRNA expression [138] and the inhibition of hepatitis B virus polymerase activity [139]. A recent study isolated a polyphenolic compound, 1,2,4,6-tetra-O-galloyl-*β*-D-glucose (1246TGG), from P. emblica, and found that treatment with 1246TGG (6.25 *μ*g/mL, 3.13 *μ*g/mL), reduced both HBsAg and HBeAg levels in culture supernatant of HepG2.2.15 cells [140]. The role of Phyllanthus spp. in the treatment of chronic hepatitis B was studied in several reports that were evaluated in a recent Cochrane review. The authors included a total of 16 randomized trials but only one compared Phyllanthus with placebo and found no significant difference in HBeAg seroconversion after the end of treatment or followup. Fifteen trials compared Phyllanthus plus an antiviral drug like interferon alpha, lamivudine, adefovir dipivoxil, thymosin, vidarabine, or conventional treatment with the same antiviral drug alone and found that the combined treatment affect serum HBV DNA, serum HBeAg, and HBeAg seroconversion. The authors conclude that Phyllanthus in combination with an antiviral drug may be better than the same antiviral drug alone but clinical trials with large sample size and low risk of bias are needed to confirm these findings [141]. 

The metanoic extracts of P. amarus root and leaf are also recently studied for the treatment of chronic hepatitis C and the root extract showed significant inhibition of HCV-NS3 protease enzyme; whereas the leaf extract showed considerable inhibition of NS5B in the *in vitro* assays. Both extracts significantly inhibited replication of HCV monocistronic replicon RNA and HCV H77S viral RNA in HCV cell culture system. Furthermore, addition of root extract together with IFN-*α* showed additive effect in the inhibition of HCV RNA replication [142]. 

### 7.4. Phyllanthus, Prevention, and Treatment of Liver Cancer

P. emblica and P. urinaria are Phyllanthus species most studied for cancer treatment. 

Water extract of P. urinaria induces apoptosis by DNA fragmentation and increased caspase-3 activity, reduces the viability of numerous cancer cells lines probably by telomerase suppression activity, and reduces the angiogenesis as suppressing MMP-2 secretion and inhibiting MMP-2 activity through zinc chelation [143]. 

P. emblica and some of its phytochemicals such as gallic acid, ellagic acid, pyrogallol, some norsesquiterpenoids, corilagin, geraniin, elaeocarpusin, and prodelphinidins B1 and B2 possess antineoplastic effects. It also possess other properties that are efficacious in the treatment and prevention of cancer as radiomodulatory, chemomodulatory, chemopreventive effects, free radical scavenging, antioxidant, anti-inflammatory, antimutagenic, and immunomodulatory activities [144]. 

In liver, P. emblica and P. urinaria inhibited HepG2 cell growth and five other cancer cell lines [145, 146]. Progallin A isolated from the acetic ether part of the leaves inhibited the proliferation of BEL-7404 cells, upregulated Bax, and downregulated Bcl-2 expression [147]. Defatted methanolic fruit extract of P. emblica suppressed carcinogen-induced response in rat liver with diethylnitrosamine-induced hepatocarcinoma [148]. 

## 8. LIV.52

Liv.52 is an Ayurvedic medicine that was used for 50 years in in the prevention and treatment of viral hepatitis, alcoholic liver disease, early cirrhosis, and a variety of conditions as protein energy malnutrition, loss of appetite, and others. It is composed by *Capparis spinosa, Cichorium intybus, Mandur bhamsa, Solarium nigrum, Terminalia arjuna, Cassia occidentalis, Achillea millefolium*, and *Tamarix gallica* [149]. 

The potential cytoprotective effect of Liv.52 was studied *in vitro* studies: it improved copper [150] and tert-butyl hydroperoxide (t-BHP) [151] toxicity in HepG2 cells by inhibition of lipid peroxidation, and increase of GSH content and antioxidant enzyme activity. Another recent study found that Liv.52 abrogated the ethanol-induced PPAR*γ* suppression and ethanol-induced TNF*α* gene expression, it also upregulated PPAR*γ* mRNA [152]. Pretreatment with low (2.6 mL/kg/day) and higher doses (5.2 mL/kg/day) of Liv.52 reversed paracetamol-induced liver toxicity in mice [153, 154]. 

Few randomized controlled clinical trials were made and results were conflicting [155], recently, a double-blind, placebo-controlled study reported that cirrhotic patients treated for 6 months with Liv.52 had significantly better Child-Pugh score, decreased ascites, and decreased serum ALT and AST levels compared with placebo group [156]. 

## 9. Complementary Alternative Medicine and Hepatocellular Carcinoma

Hepatocellular carcinoma (HCC) is one of the most frequent cancers in the world and its incidence has been increasing recently in countries including the United States of America, western Europe, and eastern Asia [157]. Systemic chemotherapy plays a palliative role while yields unsatisfactory response rates, which is partly due to the poor selectivity and low uptake efficiency of chemotherapeutic drugs in tumor [158].

Since the prognosis of cirrhotic patients seems to be largely influenced by the development of HCC, every attempt should be performed to prevent HCC in such a high-risk group. Oka et al. reported in a randomized controlled trial that a kind of medicinal herb, “Sho-saiko-to” could significantly decrease hepatic carcinogenesis rate in patients wiith cirrhosis [159]. Moreover, a number of clinical and laboratory studies have been done in the past decades in order to provide the scientific basis for the effectiveness of traditional Chinese medicine against cancer. However, actually, there are a number of contradictory reports due to various factors, as inconsistency in treatment schemes, limited sampling sizes and lack of quality assurance of the herbal products well-designed randomized controlled trials (RCT). In general, most of the published clinical studies are trials without rigorous randomization or they involved single group pre-post, cohort, time series, or matched case-control studies [160]. Herbs are generally used in combination as “formulas,” in the belief that in this way their benefits were enhanced and side effects reduced. Moreover, practitioners can adjust or customize the formulas to suit individual cancer patients. Through synergistic interactions between different effective ingredients, the herbal preparation can exert its effects in several ways: (i) they can protect the noncancerous cells and tissues in the body from the possible damage caused by chemo/radiotherapy; (ii) they can enhance the potency of chemo/radiotherapy; (iii) they can reduce inflammatory and infectious complications in the tissues surrounding the carcinoma; (iv) they can enhance immunity and body resistance; (v) they can improve general condition and quality of life; (vi) they can prolong the life span of the patients in the late stages of cancer [161].

The anticancer herbal drugs can be divided into three categories based on their target: (i) drugs that uniquely target topoisomerases (Topos) and perturb DNA replication; (ii) drugs that kill tumor cells through apoptotic pathways; (iii) drugs that alter signaling pathway(s) required for the maintenance of transforming phenotypes of the tumor cells. The cellular and animal studies have provided strong molecular evidences for the anticancer activities of the herbal medicine; however, several important questions remain to be answered. Specifically, three specific issues that will require focused attention: (i) more well-designed clinical trials to support the effectiveness and the safety of TCM in the management of cancers; (ii) new parameters based on the unique properties and theory of TCM to assess the clinical efficacy of TCM in clinical trials; (iii) new approaches to research, given the nature of TCM herbs as being fundamentally different from drugs. Undoubtedly, a clinical study of TCM treatment is more difficult and complicated than the study of single compound drugs. In addition, the effects, as well as the toxicity, of individual herbs or single compounds derived from the herb cannot completely reflect the benefits and toxicity of the herbal combination [162]. As a goal, to develop TCM into rational cancer therapy, more well-designed intensive clinical evaluations and translational laboratory studies are absolutely needed. Also, close collaboration between TCM and conventional Western medicine professions and a combination of TCM with modern multidisciplinary cutting-edge technologies, such as omic methodology on systems biology [163], would provide us with an attractive and effective strategy to achieve this goal.

Although there are many therapeutic strategies including chemotherapy to treat cancer, high systemic toxicity and drug resistance limit the success full outcomes in most cases. Accordingly, several new strategies are being developed to control and treat cancer. One approach could be a combination of and effective phytochemicals with chemotherapeutic agent, which, when combined, would enhance efficacy and reduce toxicity to tissues [164].

Several herbs and plants with different pharmacological properties are known to be rich of sources of chemical constituents that may have a potential for the prevention and the treatment of several human cancers. 

### 9.1. Curcumin

Curcumin has been shown with chemopreventive and chemotherapeutic properties against tumors in animal models and clinical trials [165–167]. The anticancer effects of curcumin have been documented in many cancers; it induces apoptosis through the death receptor mediated pathway and mitochondrial dysfunction and also induces DNA damage response by cleaving caspase-3. In addition, curcumin induces cell cycle arrest by downregulating the protein expression on cdc2 and inhibits the proliferation of human hepatocellular carcinoma J5 cells in a time- and dose-dependent manner.

### 9.2. Glycyrrhizin

Glycyrrhizin has been shown to successfully prevent the occurrence of primary HCC in patients with HCV-related chronic liver disease by unknown mechanisms [168]. One of the principal roles of long-term administration of glycyrrhizin in decreasing the carcinogenesis rate seemed to be anti-inflammatory ones, which would retrieve an active carcinogenic process. 

### 9.3. Quercetin

It has been shown that quercetin inhibits the growth of hepatoma cells in dose- and time-dependent manners. Particularly, in a recent study, quercetin treatment of hepatoma cells resulted in changes of cell cycle, reducing HCC progression [169]. 

Most of studies involving quercetin and HCC analyzed cotreatment with different chemotherapeutics. 

A study showed that BB-102 (a recombinant adenovirus vector expressing the human p53, GM-CSF and B7-1 genes) and quercetin synergetically suppress HCC cell proliferation and induce HCC cell apoptosis, suggesting a possible use as a combined anticancer agent [170]. 

In a different study, the authors explored the effect of combination treatment of quercetin in combination with roscovitine in hepatoma cells. Results showed that roscovitine in combination with quercetin can be considered as a potential therapeutic target for treatment of HCC [171].

Furthermore, it has been demonstrate that reactive oxygen species production is involved in quercetin-induced apoptosis in human HCC cell lines so quercetin induces favorable changes in the antioxidant defense system of hepatoma cells that prevent or delay conditions which favor cellular oxidative stress [172].

Otherwise, quercetin, by inducing oxidative stress, potentiates the apoptotic action of 2-methoxyestradiol in human hepatoma cells [173].

### 9.4. Silymarin

The chemopreventive effect of silymarin on HCC has been established in several studies using *in vitro* and *in vivo* methods; it can exert a beneficial effect on the balance of cell survival and apoptosis by interfering cytokines. In addition, anti-inflammatory activity and inhibitory effect of silymarin on the development of metastases have also been detected. In some neoplastic diseases, silymarin can similarly be administered as adjuvant therapy.

### 9.5. Phyllanthus

Phyllantus Emblica exhibits a variety of pharmacological effects including antiinflammatory, antipyretic, antioxidant and anti-mutagenic effects [174]. The active principles of extracts of P. emblica have demonstrated anti-proliferative effects in several cancer cell lines both *in vivo* and *in vivo*, thanks to their ability to interfere with cell cycle regulation via the inhibition of cdc 25 phosphatase and partial inhibition of cdc 2 kinase activity [175].

A study examined the growth inhibitory effect of P. emblica on human hepatocellular carcinoma (HepG2) and its synergistic effect with doxorubicin and cisplatin: the effect of chemotherapeutic agents may be modified by combination of P. emblica and be synergistically enhanced in some cases [176]. Depending on the combination ratio, the doses for each drug for a given degree of effect in the combination may be reduced. The mechanism involved in this interaction between chemotherapeutic drugs and plant extracts remains unclear and should be further evaluated.

## 10. Herbal Products and Their Side Effects

Although research on complementary and alternative medicine (CAM) therapies is still limited, this systematic review has revealed sufficient evidence to conclude that CAM, particularly the herbal products examined can be effective for certain conditions. There are reliable evidences of potential therapeutic benefit. At the same time, the more limited state of knowledge regarding the side effects of this herbal products are studied in this issue. 

These “natural products” have multiple pharmacological actions on various human physiological systems that would support the treatment of chronic disease like cancer. Moreover, the use of herbal medicines is safe compared with synthetic drugs. Further studies are required to determine the molecular mechanisms of their active ingredients.

The limitations of available clinical trials with regard to establishing safety are the same as those for establishing efficacy.

Several studies remark the importance of their protective effects for their principle antioxidants effects useful because it may help to prevent carcinogenicity-associated proliferative processes, but there are not recent publication about their toxicity or their side effects derived by their cronic or acute use. Anyway, if it presents, the side effects are poor (i.e., Glycyrrizin can induce hypokalemia, sodium retention, increase in body weight, and elevated blood pressure) [177].

Finally, hepatic damage from conventional drugs is widely acknowledged and most physicians are well aware of them. It is important to remember that acute and/or chronic liver damage occurred after ingestion of some Chinese herbs, herbals that contain pyrrolizidine alkaloids, germander, greater celandine, kava, atractylis gummifera, callilepsis laureola, senna alkaloids, chaparral, and many others. Several herbals have been identified as a cause of acute and chronic hepatitis, cholestasis, drug-induced autoimmunity, vascular lesions, and evenhepatic failure [79] (Table 1).

## 11. Conclusions 

Oxidative stress is the common pathway of chronic liver diseases of different etiology (both viral and alcoholic). CAM seems to exert an antioxidant and antifibrotic effect on liver (even if histological proof of these actions is not provided in all studies), so its use alone or in association with etiologic and causal standard therapies is actually common. 

For the majority of herbal products, proof of efficacy by randomized, placebo-controlled clinical trials is often lacking. Anecdotal success and personal experience are frequently the driving force for acceptance of CAM in the population [178]. 

In contrast to pharmaceuticals, CAM are usually distributed as “food supplements” and not evaluated formally for safety and efficacy; variations in methods of harvesting, preparing, and extracting the herb, which can result in dramatically different levels of certain alkaloids. The biologically active substances have been structurally defined and standardized for only a few of the herb: in most countries, their use is neither regulated nor controlled [179]. 

It has been clearly shown that herbal products can protect the liver from oxidative injury, promote virus elimination, block fibrogenesis, or inhibit tumor growth, but the active molecules must be isolated and tested in suitable culture and animal experiments and finally in randomized, placebo-controlled studies to enable rational clinical use of the agents [180].

## Figures and Tables

**Table 1:** Some herbal drugs associated with liver damage.

Herbal	Application	Toxicity (clinical presentation)
Atractylis gummifera	Antiemetic, diuretic, chewing gum	Acute hepatitis, FHF (fulminant hepatic failure)
Callilepsis laureola	Miscellaneous	Like Atractylis gummifera
Chaparra	Antioxidant, liver and health tonic, snake bites	Cholestasis, cholangitis, chronic hepatitis, cirrhosis
Greater Celandine	Dyspepsia, irritable bowel syndrome	Chronic (cholestatic) hepatitis, fibrosis
Germander Teucrium chamaedrys	Weight reduction	Acute and chronic hepatitis, fibrosis (subacute forms)
Kava	Anxiolytic, sleeping aid	Acute and chronic hepatitis, cholestasis, FHF
Pyrrolizidine alkaloids (PA)	Herbal tea, contamination of flour	Veno-occlusive disease
Sassafras	Herbal tea	Hepatocarcinogenesis (animals)
Valerian	Sedative	Mild hepatitis
